# MicroRNA-320a inhibits breast cancer metastasis by targeting metadherin

**DOI:** 10.18632/oncotarget.9572

**Published:** 2016-05-24

**Authors:** Juan Yu, Ji-Gang Wang, Lei Zhang, Hai-Ping Yang, Lei Wang, Di Ding, Qi Chen, Wen-Lin Yang, Ke-Han Ren, Dan-Mei Zhou, Qiang Zou, Yi-Ting Jin, Xiu-Ping Liu

**Affiliations:** ^1^ Department of Pathology, School of Basic Medical Sciences, Fudan University, Shanghai 200032, China; ^2^ Department of Pathology, The Affiliated Hospital of Qingdao University, Qingdao 266003, China; ^3^ Department of Pathology, People's Hospital, Linzi District, Zibo 255400, China; ^4^ Department of Breast Surgery, Huashan Hospital, Fudan University, Shanghai 200040, China; ^5^ Department of Pathology, The Fifth People's Hospital, Fudan University, Shanghai 200240, China

**Keywords:** miR-320a, MTDH, metastasis, breast cancer

## Abstract

Dysregulated microRNAs play important pathological roles in carcinogenesis that are yet to be fully elucidated. This study was performed to investigate the biological functions of microRNA-320a (miR-320a) in breast cancer and the underlying mechanisms. Function analyses for cell proliferation, cell cycle, and cell invasion/migration, were conducted after miR-320a silencing and overexpression. The specific target genes of miR-320a were predicted by TargetScan algorithm and then determined by dual luciferase reporter assay and rescue experiment. The relationship between miR-320a and its target genes was explored in human breast cancer tissues. We found that miR-320a overexpression could inhibit breast cancer invasion and migration abilities *in vitro*, while miR-320a silencing could enhance that. In addition, miR-320a could suppress activity of 3′-untranslated region luciferase of *metadherin* (*MTDH*), a potent oncogene. The rescue experiment revealed that *MTDH* was a functional target of miR-320a. Moreover, we found that *MTDH* was negatively correlated with miR-320a expression, and it was related to clinical outcomes of breast cancer. Further xenograft experiment also showed that miR-320a could inhibit breast cancer metastasis *in vivo*. Our findings clearly demonstrate that miR-320a suppresses breast cancer metastasis by directly inhibiting *MTDH* expression. The present study provides a new insight into anti-oncogenic roles of miR-320a and suggests that miR-320a/MTDH pathway is a putative therapeutic target in breast cancer.

## INTRODUCTION

MicroRNAs (miRNAs) are a class of evolutionally conserved, small (18-25 nucleotides), non-coding RNAs that have an important function in post-transcriptional gene regulation [1]. More recent identifications reveal that hundreds of miRNAs are aberrantly expressed in cancerous tissues through high-throughput biochemical screens [2, 3]. Unfortunately, lack of further studies to clearly define their molecular roles has resulted in that these miRNAs are only “cancer related”.

Cancer can have severe health consequences, and is a leading cause of death. Breast, colorectal, lung, uterine cervix, and stomach cancer are the most common types of cancer among women [4, 5]. MicroRNA-320a (miR-320a) has been reported to be deregulated in multiple types of cancers, including intrahepatic cholangiocarcinoma [6], colon cancer [7], primary squamous cell lung cancer [8], and prostate cancer [9]. Available data suggest that miR-320a plays pivotal roles in key cellular processes of carcinogenesis [10, 11]. To date, only a few target genes of miR-320a are validated, including *β-catenin* [7], p*olycomb complex protein Bmi-1* [10], *integrin β3* [12], i*nsulin-like growth factor-1 receptor* [13], *small GTP binding protein Rac1* [14], *ARPP-19* [15], *survivin* [16], and *neuropilin-1* [17], etc. More candidate target genes of miR-320a still need to be further explored.

*Metadherin* (*MTDH*) is reported as a neuropathology-associated gene produced in human fetal astrocytes following HIV-1 infection or treatment with recombinant HIV-1 envelope glycoprotein [18]. Human *MTDH* is located at chromosome 8q22 and encodes a 582-amino acid protein. It is consistently found to be overexpressed in breast cancers, and is significantly correlated with breast cancer progression and clinical outcomes [19]. Just recently, several reports reveal that *MTDH* is regulated by microRNAs and involved in cancer development [20–23].

Our earlier study showed that miR-320a expression in breast cancers with lymph node (LN) metastasis was significantly lower than those without LN metastasis, which indicated that miR-320a was involved in the process of breast cancer metastasis [24]. However, the detailed biological roles of miR-320a in breast cancer and the underlying mechanisms remain unexplored. In the present study, we investigated the *in vitro* and *in vivo* biological functions of miR-320a, and then tried to identify its potential targets. We examined miR-320a expression in 41 pairs of fresh breast cancer and their corresponding non-tumorous breast tissues, which showed that miR-320a was significantly downregulated in breast cancer tissues. Luciferase reporter assay and further rescue study revealed that *MTDH* was the functional target gene of miR-320a. Decreased miR-320a expression was associated with high MTDH expression which contributed to breast cancer metastasis and poor prognosis.

## RESULTS

### MiR-320a is downregulated in breast cancer tissues and cell lines

**Figure 1:**
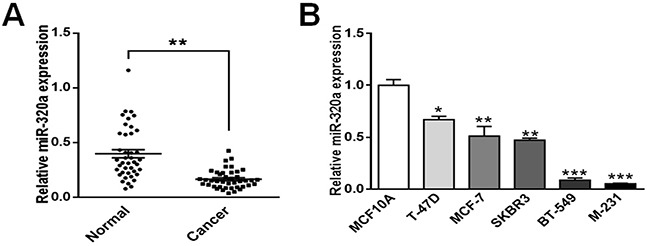
MiR-320a expression in breast cancer tissues/cells and normal breast tissues/cells **A.** miR-320a was underexpressed in breast cancer tissues. Data were analyzed by student's t test. **B.** miR-320a was underexpressed in breast cancer cell lines (MDA-MB-231, BT-549, SK-BR-3, MCF-7, and T-47D) compared with non-tumorigenic breast epithelial cell line MCF10A. MDA-MB-231 cells and BT-549 cells, which appeared more spindle, showed the lowest expression levels. Data were analyzed by Dunnett's multiple comparison. Vertical bars indicate SEM. *, *P* < 0.05 vs. MCF10A; **, *P* < 0.01 vs. MCF10A; **, *P* < 0.001 vs. MCF10A.

Our previous CISH study for miR-320a showed that miR-320a expression in invasive breast cancer with LN metastasis was significantly lower than that of breast cancer in situ, and patients exhibiting low miR-320a expression levels had shorter overall survival times. The data suggest that dysregulation of miR-320a may be involved in invasive breast cancer progression, and miR-320a presents a potential biomarker for the prognosis of invasive breast cancer [24]. To further explore miR-320a characteristics in breast cancer, we applied TaqMan qRT-PCR to quantify miR-320a expression in 41 pairs of fresh breast cancer tissues and the corresponding non-tumorous breast tissues, which showed that miR-320a was significantly lower in breast cancer tissues (*P* < 0.001) (Figure 1A). Moreover, the qRT-PCR analysis revealed that miR-320a expression level in breast cancer cell lines (T-47D, MCF-7, SK-BR-3, BT-549, MDA-MB-231) was markedly lower than MCF10A, a non-tumorigenic breast epithelial cell line. The lowest expression level was observed in BT-549 and MDA-MB-231 cells, which appeared more spindle and capable of metastasizing (Figure 1B). In terms of these findings, we hypothesize that miR-320a might act as a tumor suppressor in breast cancer.

### MiR-320a suppresses migration and invasion of human breast cancer cells

**Figure 2:**
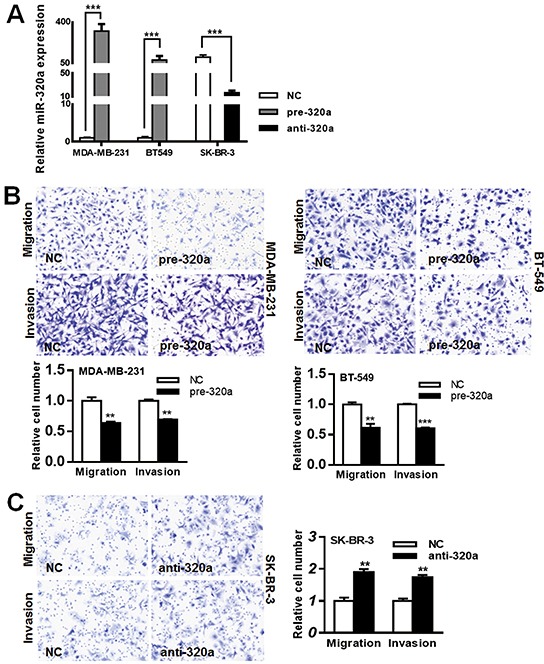
MiR-320a inhibited breast cancer cell migration and invasion **A.** The transfection efficiencies for pre-miR-320a and anti-miR-320a were validated by qPCR. After transfection for 24 h, total RNAs were extracted and PCR for miR-320a was performed. Pre-miR-320a transfection in MDA-MB-231 and BT-549 cells significantly upregulated miR-320a expression (more than 50 folds), and anti-miR-320a transfection in SK-BR-3 cells significantly downregulated miR-320a expression (about 5 folds). **B.** Migration/invasion inhibition in MDA-MB-231 and BT-549 cells after pre-miR-320a transfection. Cells were seeded in chambers after transfection for 24 h. After seeding for 18 h and 36 h, cells pass through the membrane were calculated respectively for migration assay and invasion assay. The results showed that the passed cells significantly decreased after pre-miR-320a transfection. **C.** Migration/invasion promotion in SK-BR-3 cells after anti-miR-320a transfection. After seeding for 24 h and 48 h, cells passed through the membrane were calculated respectively for migration assay and invasion assay. Anti-miR-320a transfection resulted in a ~2-fold increasement of passed cells for migration assay and a ~1.7-fold increasement for invasion assay. The value of “relative cell number” was calculated by experimental group/NC group. Data were analyzed by student's t test. Vertical bars indicate SEM. **, *P* < 0.01; ***, *P* < 0.001.

Next we investigated the biological roles of miR-320a in breast cancer cells. MDA-MB-231 and BT-549 cells were transfected with pre-miR-320a, and SK-BR-3 cells were transfected with anti-miR-320a. The PCR results demonstrated that pre-miR-320a transfection could significantly upregulate miR-320a expression in MDA-MB-231 and BT-549 cells (more than 50 folds), and anti-miR-320a transfection could significantly downregulate miR-320a expression in SK-BR-3 cell (about 5 folds) (Figure 2A). Thereafter, we performed CCK8 proliferation assay and transwell migration/invasion assay to investigate the *in vitro* biological functions of miR-320a. We found that miR-320a upregulation in MDA-MB-231 and BT-549 cells significantly inhibited cell migration and invasion abilities (Figure 2B, Supplementary Figure S1), and miR-320a downregulation in SK-BR-3 cells significantly promoted cell migration and invasion (Figure 2C, Supplementary Figure S1). However, miR-320a alteration could not affect proliferation of breast cancer cells (Supplementary Figure S2).

### MiR-320a directly regulates MTDH and VDAC1

MiRNAs usually exert their functions by negatively modulating the expression of target genes. To explore the possible mechanisms implicated in the suppression of migration and invasion induced by miR-320a, we adopted a widely acknowledged bioinformatics tools (TargetScan) to search for the potential targets. More importantly, we used the online Kaplan-Meier Plotter analysis (http://www.kmplot.com/breast) to narrow down the targets within potential oncogenes, and finally identified six genes (*GNB2L1*, *GRB2*, *HLTF*, *MTDH*, *VDAC1* and *YWHAZ*) which were among the possible target mRNAs of miR-320a.

**Figure 3:**
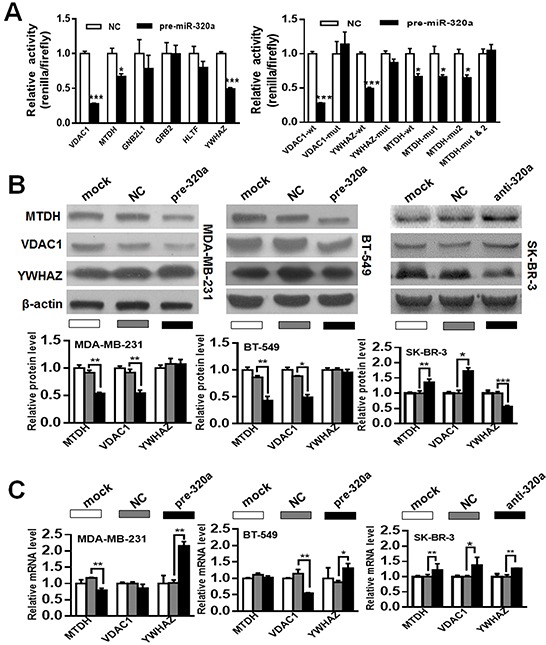
MiR-320a targeted *VDAC1* and *MTDH* in breast cancer cells **A.** Dual luciferase activity in 293T cells upon co-transfection of wild-type (wt) or mutant (mt) 3′-UTR-driven reporter construct and pre-miR-320a. We found that luciferase activity was decreased by pre-miR-320a in three (*MTDH*, *VDAC1* and *YWHAZ*) of the six wt-3′UTR vectors compared with pre-NC, whereas mutation of the binding sites in these 3′UTR-containing vector abolished responsiveness to pre-miR-320a. Data were analyzed by student's t test. *, *P* < 0.05; ***, *P* < 0.001. **B, C.** *MTDH*, *VDAC1* and *YWHAZ* protein (B) and mRNA expressions (C) in breast cancer cells after miR-320a introduction and suppression. MiR-320a alteration could significantly affect *MTDH* and *VDAC1* expressions, but not *YWHAZ.* Data were analyzed by Dunnett's multiple comparison. Vertical bars indicate SEM. *, *P* < 0.05 vs. NC; **, *P* < 0.01 vs. NC; **, *P* < 0.001 vs. NC.

We cloned the wt-3′UTR of the potential target genes containing putative miR-320a binding sites into the pluc-reporter vector, and generated a series of luciferase reporter vectors. By cotransfection of pre-miR-320a and wt-pluc-reporter vector, we found that luciferase activity was decreased by pre-miR-320a in three (*MTDH*, *VDAC1* and *YWHAZ*) of the six wt-3′UTR containing vectors compared with pre-NC, whereas mutation of the binding sites in these 3′-UTR-containing vector abolished responsiveness to pre-miR-320a (Figure 3A). Next we assessed the effect of miR-320a on *MTDH*, *VDAC1* and *YWHAZ* protein levels by performing western blot. Overexpression of miR-320a in MDA-MB-231 and BT-549 cells resulted in significant reduction of MTDH and VDAC1 protein expression levels, whereas YWHAZ protein level was unchanged. In contrast, inhibition of miR-320a expression in SK-BR-3 cells resulted in marked induction of MTDH and VDAC1 protein expression, whereas YWHAZ protein level was decreased (Figure 3B). Moreover, qRT-PCR analysis revealed that miR-320a overexpression caused suppression or degradation of *MTDH* and *VDAC1* mRNA in MDA-MB-231 and BT-549 cells. However, it did lead to increased *YWHAZ* mRNA level in these two cells. Comparatively, suppression of miR-320a expression caused increased mRNA levels of *MTDH*, *VDAC1* and *YWHAZ* in SK-BR-3 cells (Figure 3C). To conclude, these results indicate that *MTDH* and *VDAC1* are potential targets of miR-320a in breast cancer.

### MTDH is a functional target of miR-320a

**Figure 4:**
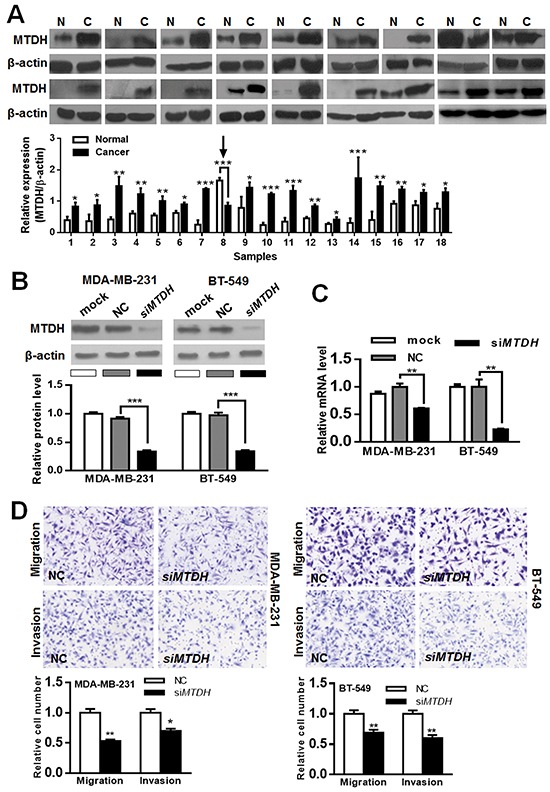
MTDH expression in paired breast cancer and normal tissue samples and its *in vitro* effect on breast cancer migration/invasion **A.** Among the 18 cancer tissues with low miR-320a expression, MTDH was overexpressed in 17 (arrow, MTDH was underexpressed in cancer tissues). **B, C.** Validation of siRNA-MTDH (siMTDH) silencing efficiency. MTDH protein (B) and MTDH mRNA (C) were significantly downregulated after siMTDH transfection. **D.** Migration and invasion inhibition after siMTDH silencing in breast cancer cells. Data were analyzed by student's t test. Vertical bars indicate SEM. *, *P* < 0.05**; *P* < 0.01; ***, *P* < 0.001.

To assess whether *MTDH* and *VDAC1* are clinically correlated with miR-320a expression, we examined their expression in 18 paired fresh tissue samples where miR-320a was underexpressed in breast cancer more than 2-fold in comparison to normal adjacent breast tissues by western blot (MTDH and VDAC1) and PCR (miR-320a). Only four cancer tissues (22.2%) showed higher VDAC1 expression (Supplementary Figure S3), while 17 cancer tissues (94.4%) showed higher MTDH expression (Figure 4A).

**Figure 5:**
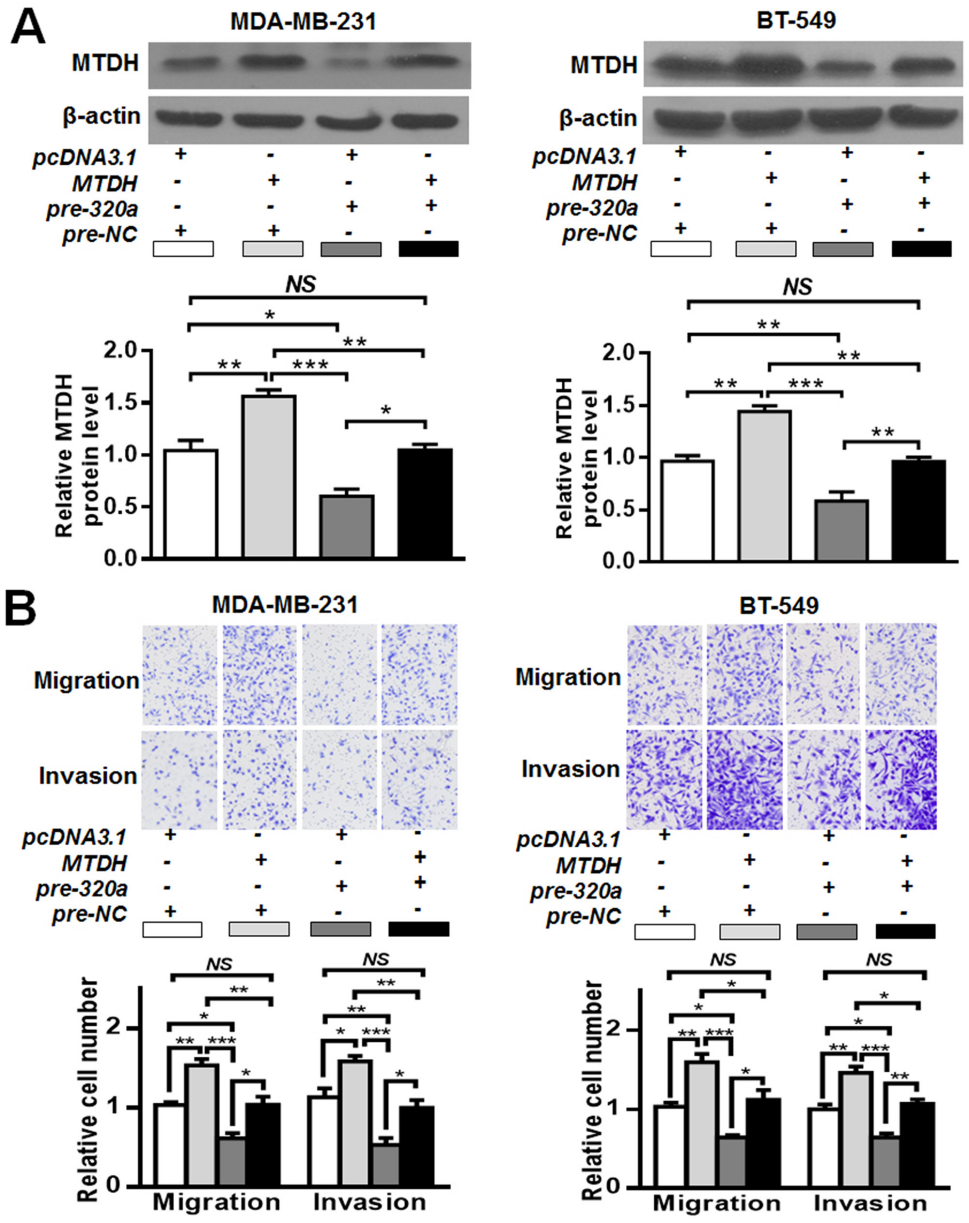
The interaction between miR-320a and MTDH **A.** MTDH alteration after pcDNA3.1-*MTDH* and/or pre-miR-320a co-transfection. MDA-MB-231 and BT-549 cells were co-transfected with pre-miR-320a and pcDNA3.1-*MTDH*. The western blot results showed that miR-320a could effectively inhibit MTDH expression. **B.** Transwell assays after pre-miR-320a and pcDNA3.1-*MTDH* co-transfection. PcDNA3.1-*MTDH* transfection could counteract miR-320a induced migration/invasion inhibition. Data were analyzed by one-way ANOVA. Vertical bars indicate SEM. *NS*, no significance; *, *P* < 0.05; **, *P* < 0.01, ***, *P* < 0.001.

Next we ascertained whether *MTDH* reduction might produce a suppression of cell invasion/migration similar to miR-320a overexpression. We found that transfection of siRNAs against *MTDH* (Figure 4B, 4C) in MDA-MB-231 and BT-549 cells reduced cell invasion and migration abilities (Figure 4D, Supplementary Figure S1), which is similar to the effect of miR-320a overexpression. Thereafter, we generated a “rescue” assay to investigate the effect of miR-320a in the presence of *MTDH* overexpression. After transfection with pcDNA3.1-MTDH or pcDNA3.1-control, pre-miR-320a or pre-NC was co-introduced into these cells (Figure 5A). The transwell experiment demonstrated that the enforced expression of *MTDH* partially restored the migratory and invasive abilities of MDA-MB-231 and BT-549 cells (Figure 5B, Supplementary Figure S1). Collectively, these findings suggest that *MTDH* is a functional target of miR-320a in breast cancer.

### MTDH status correlates with miR-320a expression in breast cancer

**Figure 6:**
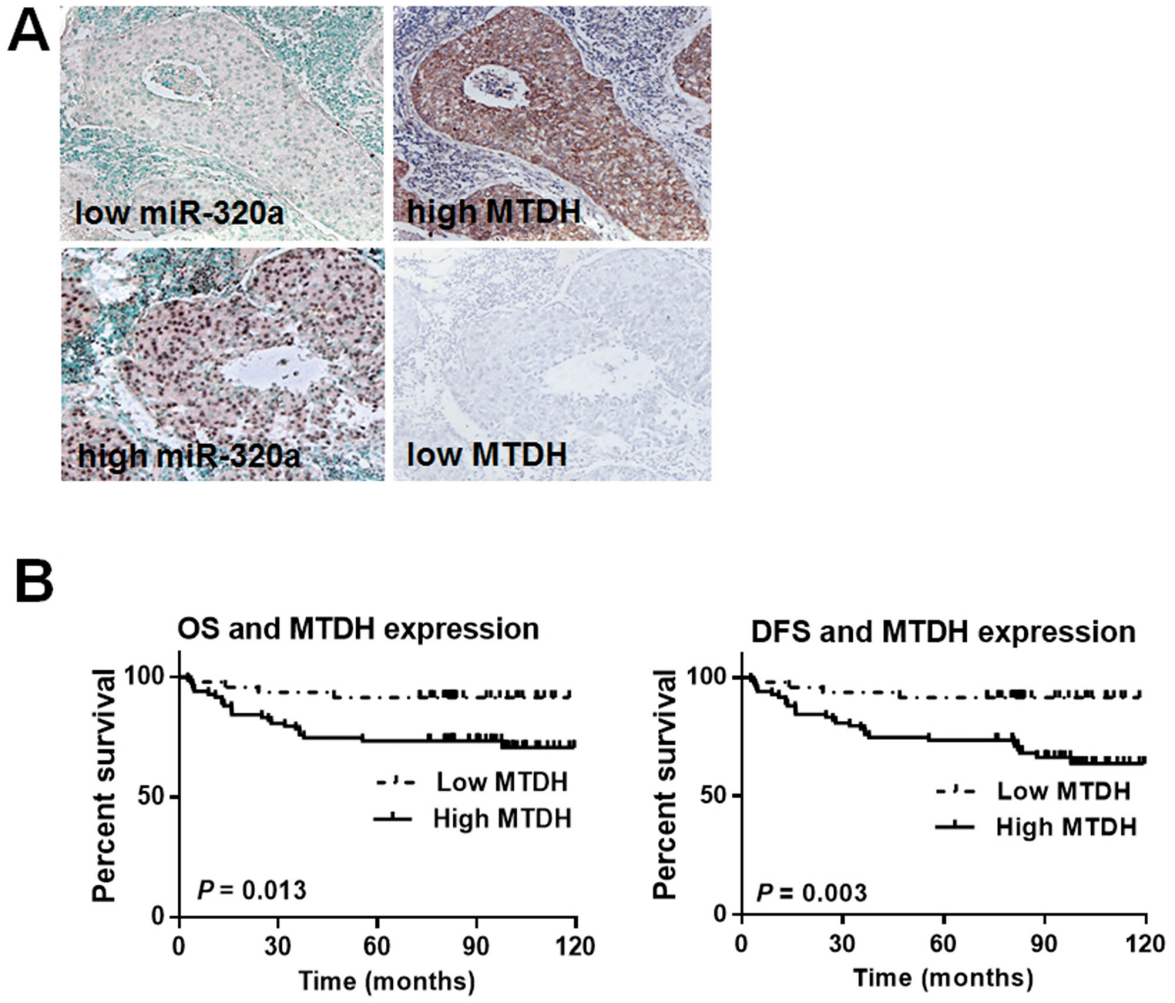
MTDH expression was inversely correlated with the expression level of miR-320a **A.** Representative images of MTDH and miR-320a expression in consecutive sections. MTDH expression was inversely correlated with the expression level of miR-320a. Immunohistochemistry was performed for MTDH, and CISH was performed for miR-320a. **B.** Kaplan-Meier survival curves in 130 invasive breast cancer patients with different MTDH levels. A total of 83 cases of breast cancer tissue showed low expression of MTDH, and 47 cases showed high expression. During the follow-up period, a total of 28 patients with high MTDH expression suffered recurrence/metastasis and 23 died of cancer related complications. In contrast, only 4 patients with low MTDH expression suffered cancer recurrence/metastasis and vanished. Further log-rank test showed that MTDH expression level affected OS and DFS patterns. Patients with high MTDH expression suffered a short life expectancy and a high recurrence/metastasis possibility.

To further assess the relationship between miR-320a and MTDH, the expression of MTDH was investigated in 130 invasive breast cancer FFPE samples using IHC. MTDH immunoreactivity was mainly detected in the cytoplasm (Figure 6A), and 83/130 (63.8%) showed high expression of MTDH (Table 1). When taking the CISH result for miR-320a into account together with the present IHC result for MTDH, we found an inverse relationship between miR-320a and MTDH (*P* = 0.007) (Table 1, Figure 6A). These data indicated that MTDH overexpression might be attributed to miR-320a reduction in breast cancer.

**Table 1:** MiR-320a and MTDH expression level in 130 breast cancer samples

miR-320a expression	MTDH expression	
	High	Low
High	29	31
Low	54	16

The chi-square test showed there existed an inverse relationship between miR-320a and MTDH expressions (*P* = 0.007).

### MTDH expression is related to breast cancer progression

To evaluate the clinical significance of MTDH expression in breast cancer, we analyzed the relationship between MTDH expression and the clinicopathologic characteristics. As is shown in Table 2, MTDH expression was associated with tumor size (*P* = 0.016), LN metastasis (*P* = 0.02) and clinical TNM stage (*P* = 0.049). However, MTDH expression was not related to age, menopause status, histological grade, and the expression status of estrogen receptor, progesterone receptor and HER2. Further survival analysis showed that high MTDH expression was an adverse prognostic factor for invasive breast cancer (*P* = 0.039) (Figure 6B).

**Table 2:** Correlation of MTDH expression and the clinicopathologic characteristics in invasive breast cancer (cases)

Characteristics	n	MTDH expression		*P* value
		High expression	Low expression	
**Age (years)**				0.636
< 45	15	8	7	
45-55	61	39	22	
> 55	54	36	18	
**Menopause**				0.686
No	50	33	17	
Yes	80	50	30	
**Tumor size (cm)**				0.016
≤ 2.5	79	44	35	
> 2.5	51	39	12	
**Lymph node metastasis**				0.02
0	71	39	32	
1-2	32	21	11	
>2	27	23	4	
Histological grade^*^				0.801
Grade I	14	10	4	
Grade II	93	59	34	
Grade III	23	14	9	
pTNM stage^*^				0.049
I	44	24	20	
II	54	33	21	
III-IV	32	26	6	
**Estrogen receptor**				0.729
Negative	61	38	23	
Positive	69	45	24	
**Progesterone receptor**				0.286
Negative	75	45	30	
Positive	55	38	17	
**HER2 status**				0.273
Negative	50	29	21	
Positive	80	54	26	
**Survival**				0.000
Yes	103	61	42	
No	27	22	5	
**Recurrence/metastasis**				0.000
Yes	32	19	13	
No	98	64	34	

* According to World Health Organization Classifications of Tumors of Breast, 4^th^ edition, 2012.

### MiR-320a inhibits breast cancer invasion and metastasis in vivo

**Figure 7:**
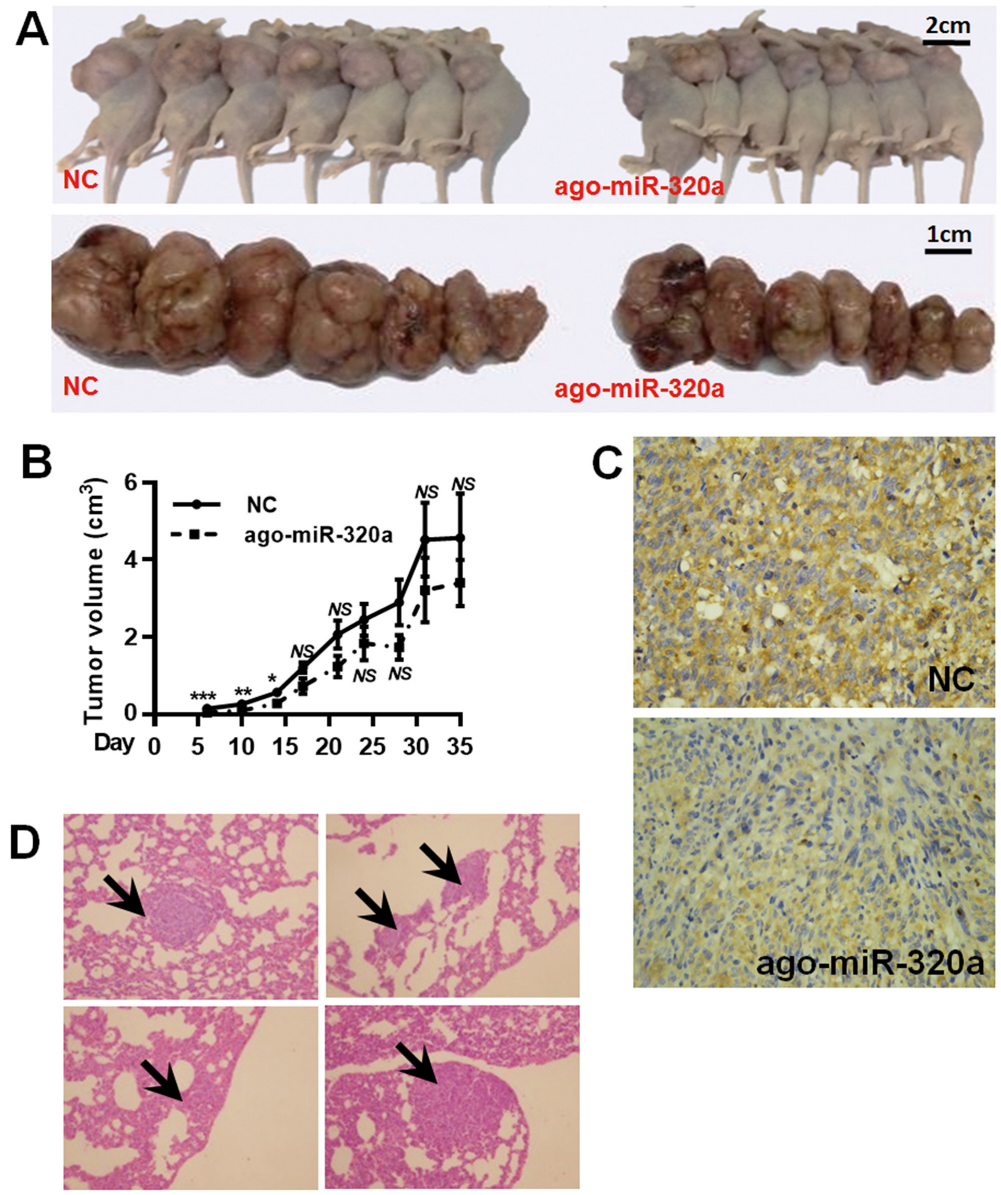
MiR-320a inhibited breast cancer metastasis *in vivo* **A.** Tumor formation after injection of MDA-MB-231 cell treated with ago-miR-320a or *NC*. **B.** The growth curves showed miR-320a could inhibit tumor growth in the first two weeks. *NS*, no significance; *, *P* < 0.05 vs. NC; **, *P* < 0.01 vs. NC; **, *P* < 0.001 vs. NC. **C.** Immunohistochemical staining for MTDH in xenograft tumors. The tumor with ago-miR-320a transfection showed weaker MTDH expression than NC tumor. **D.** Representative images of metastatic nodules in the lung of NC group (4 mice).

To further determine the *in vivo* effect of miR-320a, we treated MDA-MB-231 cells with ago-miR-320a (Supplementary Figure S4). MDA-MB-231 cells with miR-320a overexpression were subcutaneously injected into the third mammary pads. The mice displayed visible mammary tumors at week 1, and became moribund at week 5 owing to primary tumor burden. As compared with the NC group (transfected with cognate RNA), the xenograft tumors derived from MDA-MB-231 cells transfected with ago-miR-320a grew substantially slowly in the first two weeks (Figure 7A, 7B). Nonetheless, the tumor cells showed lower MTDH expression than NC group (Figure 7C). Of the 7 mice in NC group, 4 (57.1%) developed lung metastasis while none suffered metastasis in the mice with ago-miR-320a transfection (Figure 7D). Therefore, we conclude that miR-320a can suppress *MTDH* expression and inhibit breast cancer invasion and metastasis *in vivo*.

## DISCUSSION

Alteration of miRNAs is of great importance in breast cancer development [25]. Nevertheless, more miRNAs in breast cancer progression still require further exploration. Of particular importance is the identification of penitential targets of the ontogenetic miRNAs. In the present study, we firstly demonstrated that miR-320a suppressed the migration and invasion abilities of breast cancer. In addition, we found that *MTDH*, a metastasis adhesion gene that is frequently overexpressed in breast, prostate, liver, kidney and colon cancer [26], was a novel functional target of miR-320a in breast cancer. Downregulation of miR-320a would result in the *MTDH* overexpression which contributes to the progression of breast cancer.

On the basis of our earlier study [24], we examined miR-320a expression by qRT-PCR in 41 paired fresh frozen breast samples. The result showed that miR-320a was downregulated in breast cancer, which was in favor of previous report which suggested that miR-320a functions as a tumor suppressor. In the present study, we also demonstrated that miR-320a suppressed breast cancer cell migration and invasion. The *in vivo* xenograft experiment confirmed this finding. Nonetheless, we found that the tumor with higher miR-320a expression tended to grow slowly in the early stage. The volume of an *in vivo* tumor is affected by multiple factors. Apart from proliferation, local invasion ability also contributes to tumor growth. A tumor mass with stronger invasion ability is likely to invade and destroy its surrounding tissues more easily. In addition, the tumor with stronger invasion ability can easily acquire blood supply by insidiously inciting a network of blood vessels around itself. Given that miR-320a is tightly related to cancer invasion, we consider the *in vivo* tumor size is affected through miR-320a induced invasion inhibition instead of proliferation regulation.

Identification of miR-320a target genes is critical for understanding its role in carcinogenesis. Herein our data provided for the first evidence that *VDAC1* and *MTDH* are target genes of miR-320a by luciferase reporter assay. VDAC is a pore-forming protein expressed in the outer membrane of eukaryotic mitochondria. It controls the metabolic cross-talk between mitochondria and the rest of the cell, and thus influences the function of cell death and metabolism [27]. Despite the critical involvement of *VDAC1* in various tumors, little is known of *VDAC1* in breast cancer. To investigate the clinical relevance of *VDAC1*, we investigated VDAC1 expression in 18 paired breast tissues using western blot; however, only 4 cases (22.2%) suffered high expression of VDAC1, and no obvious inverse correlation was observed between miR-320a and VDAC1. More samples should be detected to further confirm these findings.

*MTDH*, the other identified target gene, is tightly relevant to carcinogenesis: it is reported to promote cancer proliferation and metastasis and be associated with poor prognosis [28–30]. In our study, the luciferase reporter assay, mRNA quantification and western blot analysis demonstrated that *MTDH* was a direct downstream target of miR-320a, and there existed an inverse relationship between miR-320a and *MTDH*. To the best of our knowledge, this is the first report to demonstrate that *MTDH* is the functional target gene of miR-320a. Overexpression of *MTDH* can activate several downstream pathways, including the Akt pathway, the nuclear factor-κB pathway, and the Wnt/β-catenin pathway, to enhance different aspects of tumor malignancy [26].

Our study showed MTDH overexpression in breast cancer predicted poor outcome. This is consistent with previous report [28]. Together with the CISH result for miR-320a expression, a significantly inverse correlation between miR-320a and MTDH can be identified (Table 1). Patients suffering LN or distant metastasis are more apt to have low miR-320a expression [24] and high MTDH expression. In addition, the function study showed *MTDH* expression was directly inhibited by miR-320a. Ectogenic overexpression of *MTDH* was able to rescue migration/invasion attenuated by miR-320a, confirming that *MTDH* was a major target of miR-320a. Moreover, function studies showed that *MTDH* silencing phenocopied overexpression of miR-320a in breast cancer cells, resulting in migration/invasion suppression. These data suggested that *MTDH* is a functional target of miR-320a. According to previous reports, miR-375, miR-26a, and miR-30a are also upstream regulating miRNAs of *MTDH* [20, 22, 31]. Further research is required to understand how these miRNAs coordinately modulate *MTDH* and their interactions, which would deepen the present knowledge about the cross-talk between miRNAs.

In conclusion, our data suggest that miR-320a may inhibit invasion and metastasis of breast cancer by downregulating MTDH. The present study provides new insight into anti-oncogenic roles of miR-320a in the breast cancer pathogenesis and suggests that miR-320a/MTDH pathway could be a putative therapeutic target in breast cancer.

## MATERIALS AND METHODS

The information of PCR, western blot, immunohistochemistry (IHC), proliferation assay, cell invasion and migration assay, and cell cycle assay is provided in the Supplementary Materials.

### Human tissue samples and cell lines

A total of 130 cases of surgical resected breast cancer samples were collected from Huashan Hospital, Fudan University. These samples were fixed in formalin and embedded in paraffin. All pathological documents, including patients' age, tumor size, lymph node status, histological grade, and pTNM stage, were carefully reviewed. The tumor dimension was measured by a pathologist when sampling, and the maximal diameter was documented and brought into this study. Follow-up data were available in all patients, with a mean follow-up of 77.5 months (range, 2.6 -120 months). None of the patients received chemotherapy or radiation therapy before surgery, and after successful radical mastectomy, all patients only received 4 cycles of cyclophosphamide, methotrexate, and 5-fiuorouracil (CMF). In addition, 23 cases of paired fresh tissue samples (breast cancer tissues and the corresponding non-tumorous breast tissues) were collected at the time of surgery and snap frozen in liquid nitrogen immediately, which were prepared for miR-320a detection. The 18 paired samples where miR-320a in cancer tissue was 2-fold lower than normal tissue prepared for determining both miR-320a and its potential targets. Ethical approval was obtained from the Clinical Research Ethics Committee, Fudan University.

The cell lines HEK-293T, SK-BR-3, T-47D, MCF-7, MDA-MB-231 and BT-549 were purchased from the cell bank of Shanghai Institute for Biological Sciences, Chinese Academy of Sciences (Shanghai, China). Cells were cultured in recommended medium supplemented with 10% fetal bovine serum (FBS, Gibco, Cat.# 10099-141, Carlsbad, CA USA). Human mammary epithelial cell line MCF10A was purchased from American Type Culture Collection (Manassas, VA USA), which was maintained in DMEM/F12 (1:1) (Gibco), supplemented with EGF (100 mg/mL) (Life Technologies, Carlsbad, CA USA), cholera toxin (100 ng/mL) (Sigma), insulin (10 mg/mL) (Sigma), hydrocortisone (1 mg/mL) (Sigma), 5% horse serum (Gibco). All cells were maintained in a humidified atmosphere with 5% CO_2_ at 37°C.

### Chromogenic in situ hybridization (CISH)

We have previously described the method for CISH [24]. Briefly, all sections were digested with pepsin, prehybridized with a prehybridization solution at 54°C for 2 h, and then hybridized at 54°C for 16–20 h with 5′-digoxin-conjugated locked nucleic acid probes for miR-320a, U6 (positive control) and scrambled RNA (negative control) (all Exiqon, Copenhagen, Denmark). After washing with Tris-buffered saline, the slides were incubated with a sheep polyclonal anti-digoxin antibody (Roche Diagnostics GmbH, Mannheim, Germany), and then stained with nitro blue tetrazolium/5-bromo-4-chloro-3-indolyl-phosphate. Methyl green was used to counterstain the nuclei. Positive results appeared blue in the cytoplasm and nuclei.

### Pre/anti-miRNA, siRNA, plasmid construction and transfection

For transient transfection, pre-miR-320a, anti-miR-320a (Invitrogen, Carlsbad, CA USA), siRNA-*MTDH* (RiboBio, Shanghai, China) and their cognate negative control (NC) RNAs were transfected into cells using lipofectamine 2000 (Invitrogen) following the manufacturer's instructions. The *MTDH* expression vector, carrying a 1952-bp human *MTDH* coding DNA sequence which was amplified by PCR, was cloned into *pcDNA3.1* vector (Invitrogen). The pcDNA3.1-*MTDH* constructs were confirmed by DNA sequencing. The primers are displayed in Supplementary Table S1. In this study, pre-miR-320a and anti-miR-320a were introduced to upregulate and downregulate miR-320a expression respectively. The transfection efficiency was validated by PCR detection after 24 h. Cell proliferation and migration/invasion assays were carried out after pre/anti-miR-320a transfection. For rescue assay, after transfection with pcDNA3.1-*MTDH* or pcDNA3.1-NC for 24 h, pre-miR-320a or pre-NC was introduced into the cells for additional 36 h, and then cells were harvested.

### Luciferase reporter assay

The wild-type 3′-untranslated sequences (wt-3′UTR) of potential targets containing the miR-320a binding site were amplified by PCR. After amplification, PCR products were synthesized and ligated to pluc-Reporter luciferase vector by Kangbio Company (Shenzhen, China). The corresponding mutated vectors (mut-3′UTR) were achieved by Fast Mutagenesis System (TransGen Biotech, Beijing, China). All constructs were verified by DNA sequencing. Synthesized PCR primers are described in the Supplementary Table S1. HEK-293T cells were seeded in 24-well plates, co-transfected with 10-nmol pre-miR-320a or pre-miR-NC and 100-ng pluc-3′-UTR, and harvested 24 hours after transfection. Luciferase activities were measured using the Dual-Luciferase Reporter Assay System (Promega, Cat.# E1910, Madison, WI USA) on a Glomax Luminometer (Promega). Renilla luciferase activity was normalized by firefly luciferase.

### In vivo xenograft experiment

The ago-miR-320a (RiboBio, Shanghai, China) for *in vivo* xenograft experiment was chemically modified and cholesterol-conjugated from a hydroxyprolinol-linked cholesterol solid support and 2′-OMe phosphoramidites [32]. After transfection with ago-miR-320a or NC for 24 h, 3×10^6^ cells were suspended in 100 μL phosphate buffered saline and then injected orthotopically into the third mammary fat pads on either side of 6 to 8-week-old female athymic nude mice (Shanghai Laboratory Animal Center, Chinese Academy of Sciences, Shanghai, China). Mice were randomized into 2 groups (7 mice each group). Tumor size was measured twice a week, and the tumor growth was analyzed by measuring tumor length (*L*) and width (*W*) and calculated with the formula π*LW*^2^/6. All mice were euthanized at the end of week 5. Xenografts and lungs were carefully dissected, and then formalin-fixed, paraffin embedded (FFPE) sections were made and stained with hematoxylin and eosin for histologic studies. All the animal work was conducted in concordance with the guidelines of the Animal Care Committee, Fudan University.

### Statistical analysis

All statistical analyses were done using SPSS 19.0 software (IBM, Armonk, NY USA). Student's t test was used to compare the difference between two groups and one-way ANOVA (including Dunnett's t test) was used to compare difference between multiple groups. Survival was calculated using the postoperative time. Overall survival (OS) and disease-free survival (DFS) were calculated using the Kaplan-Meier method and compared by log-rank test. Spearman correlation was applied to assess the correlation between miR-320*a* and *MTDH* expression. All data were presented using Prism 5.0 software (Graphpad, La Jolla, CA USA) and displayed as mean ± SEM (Standard Error of Mean). Statistical significance was *P* < 0.05.

## SUPPLEMENTARY MATERIALS AND METHODS, FIGURES AND TABLES


