# Supplementary material for: MicroRNA-320a inhibits breast cancer metastasis by targeting metadherin
Source: Oncotarget. 2016 May 24;7(25):38612–25. doi: 10.18632/oncotarget.9572 (PMC5122415; doi:10.18632/oncotarget.9572)
Supplement: Supplementary file 1 [file oncotarget-07-38612-s001.pdf]

MicroRNA-320a inhibits breast cancer metastasis by targeting metadherin

SUPPLEMENTARY MATERIALS AND METHODS

Quantitative real-time PCR (qRT-PCR)

Total RNAs were extracted from cells or tissues with Trizol reagent (Invitrogen, Carlsbad, CA USA). MiR-320a expression was determined by qRT-PCR using TaqMan miRNA Assay (Invitrogen), with *RNU6B* as an internal control. Target mRNA expression was determined by qRT-PCR with SYBR® Premix Ex Taq™ (Takara Bio, Dalian, China) with GAPDH as an internal reference. The primer sequences are listed in Supplementary Table S1. All reactions were run in ABI 7900 Fast Real Time PCR system. The Taqman miRNA assay thermal cycle condition was that one cycle at 95°C for 30 sec, followed by 40 cycles of amplification at 95°C for 5 sec, and then 60°C for 30 sec. Relative expression level was calculated by the $2^{-\Delta\Delta CT}$ method [ΔCt = Ct (miR-320a) – Ct (*GAPDH/RNU6B*), $\Delta\Delta Ct$ = ΔCt (cancer)- ΔCt (normal)], where Ct value represented the threshold cycle for each transcript.

Cell proliferation assay

Breast cancer cells were transfected with RNA oligonucleotide. Twenty-four hours after transfection, cells were seeded at a density of 5000-8000 cells per well in 96-well plates for cell proliferation assay. Cell growth was measured by the Cell Counting Kit-8 assay (Dojindo, Kumamoto, Japan) in accordance with the manufacturer's instructions. Absorbance values were tested at 450 nm on a spectrophotometer after incubating 2.5 hours.

Cell invasion and migration assay

Invasion and migration assays were performed using 24-well Transwell permeable supports with 8-µm pores (Corning Incorporated, Corning, NY USA). 1×10^5 or 5×10^4cells in serum-free media were seeded into the upper chamber with or without Matrigel (BD Biosciences, San Jose, CA USA) and the bottom chamber was filled with complete media as chemo-attractant. After several hours of incubation, cells that had invaded or migrated through the membrane were stained with crystal violet, imaged and counted by Vectra Automated Quantitative Pathology Imaging System (PerkinElmer, Waltham, MA USA).

Western blot

Tissue samples and cells were lysed in RIPA buffer containing protease inhibitor cocktail (Roche, Mannheim, Germany). Proteins were loaded and separated by electrophoresis, then transferred onto polyvinylidenedifluoride membrane by electroblotting. After blocking, membranes were incubated with primary antibodies to MTDH (1:2000 for western blot, 1:500 for immunohistochemistry, Cat. # 13860-1-AP, Proteintech, Chicago, IL USA), VDAC1 (1:1000 for western blot; Cat.# ab14734, Abcam, Cambridge, UK), YWHAZ (1:500 for western blot; Cat.# sc-1019, Santa Cruz, Dallas, TX USA), β-actin (1:1000 for western blot; Cat.# a5441, Sigma), and subsequently with secondary antibodies (1:1000 for western blot, Cat.# SA00001-1/2, Proteintech). The signals were visualized using an enhanced chemiluminescent substrate (Cat.# 34076, Thermo, Lithuania) and detected by photographic films or a FluorChem Q imaging system (ProteinSimple, Santa Clara, CA, USA).

Immunohistochemistry

Immunohistochemical staining was performed on 5-µm FFPE sections. Briefly, slides were deparaffinized, rehydrated and treated with 0.3% H_2O_2, followed by antigen retrieval and staining according to standard guidelines. Tumor cells were considered positive for MTDH when they showed cytoplasm immunoreactivity. The MTDH staining pattern was examined and scored by two independent pathologists. The scores were determined by combining the proportion of positive staining and the staining intensity over 10 visual fields a by a microscope (400×, ZEISS, Oberkochen, Germany). The staining index (SI) was calculated as: SI = staining intensity x proportion of positively stained tumor cells. Analysis in detail was described previously. Cutoff value to define the high- and low-expression of MTDH was chosen by a measurement of heterogeneity with the log-rank test statistic with respect to overall survival, and a score of 4 was chosen. Therefore, SI score ≥ 4 was taken to define tumors as high expression of MTDH and SI < 4 defined tumors as low expression.

RESULTS

MiR-320a is not involved in cell proliferation and cell cycle in breast cancer

MDA-MB-231 cells, in which miR-320a expression was relatively lower, were transfected with pre-miR-320a or pre-NC. T-47D cells with moderately higher expression of miR-320a were transfected with anti-miR-320a of anti-NC. Transfection with pre-miR-320a or anti-mir-320a led to significant increase or decrease in miR-320a expression when compared to their cognate NC as described before (Figure 2A). As is shown in Supplementary Figure S2, the cell proliferation and cell cycle analysis revealed that overexpression of miR-320a could not suppress cell proliferation or alter cell cycle distribution in MDA-MB-231 cells; meanwhile, suppression of miR-320a could not affect cell growth and cell cycle in T-47D cells. We consider miR-320a is not involved in breast cancer cell proliferation and cell cycle.

The results of migration assay for the five breast cancer cell lines (T-47D, MCF-7, SK-BR-3, BT-549, MDA-MB-231) are shown in Supplementary Figure S5.

SUPPLEMENTARY FIGURES AND TABLES

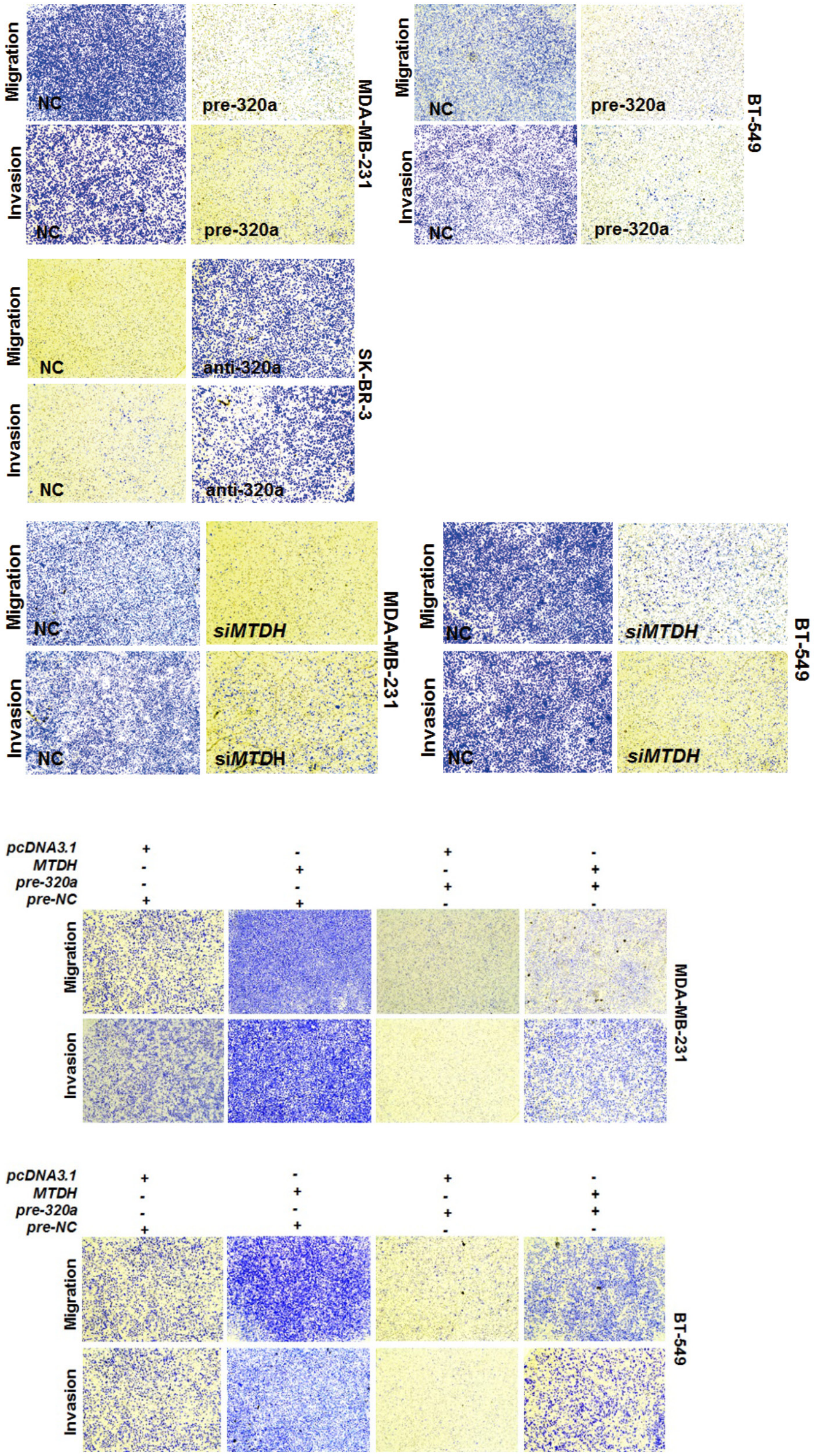

Supplementary Figure S1: Low power microscopic graphs of migration and invasion assay (original magnification, 40 ×).

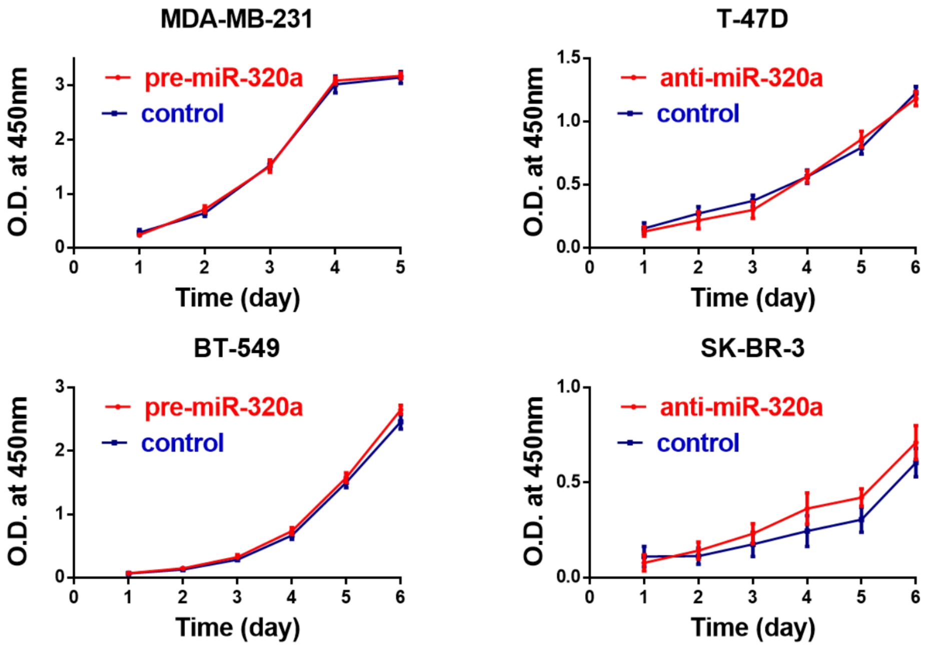

Supplementary Figure S2: MiR-320a could not affect proliferation in breast cancer cells. Pre-miR-320a was introduced in MDA-MB-231 cells to upregulate miR-320a expression, and anti-miR-320a was introduced in T-47D and SK-BR-3 cells to downregulate miR-320a expression. CCK8 assay was performed in the following days to detect the effect of miR-320a on cell proliferation. We found that the differences between treated groups and control groups at each day were not significant. Data were analyzed using student's t test. Vertical bars indicate SD.

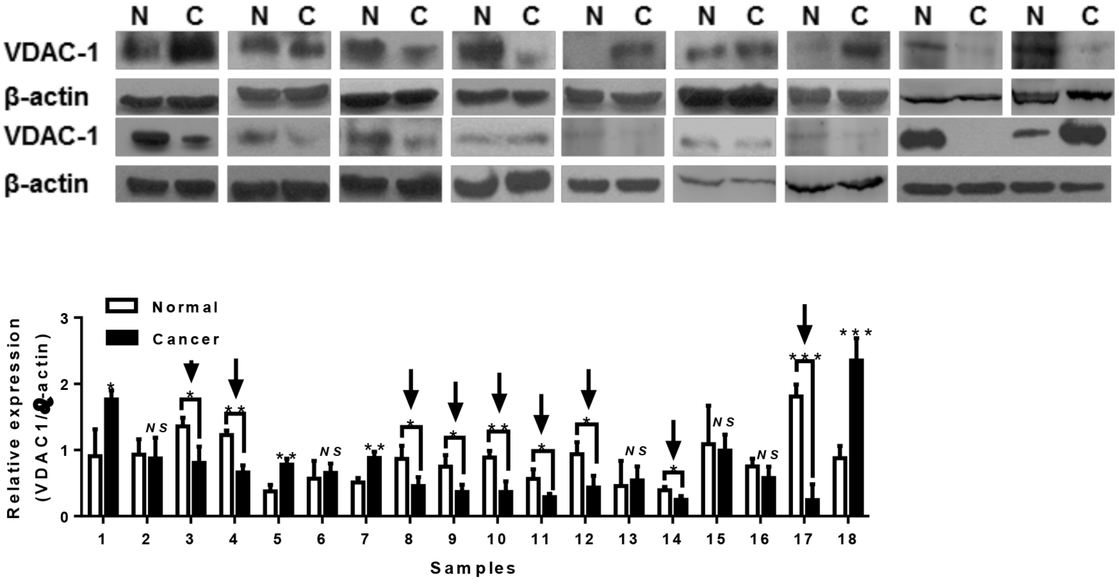

Supplementary Figure S3: VDAC1 expression in 18 paired breast cancer and adjacent normal breast tissues (N, non-cancerous tissue; C, cancer tissue). Only 4 cases of cancer tissues showed higher expression for VDAC1 than normal tissues, while 9 cases showed lower expression for VDAC1 (arrows). Data were analyzed by student's t test. Vertical bars indicate SEM. *, $P < 0.05$**; $P < 0.01$; ***, $P < 0.001$; *NS*, no significance.

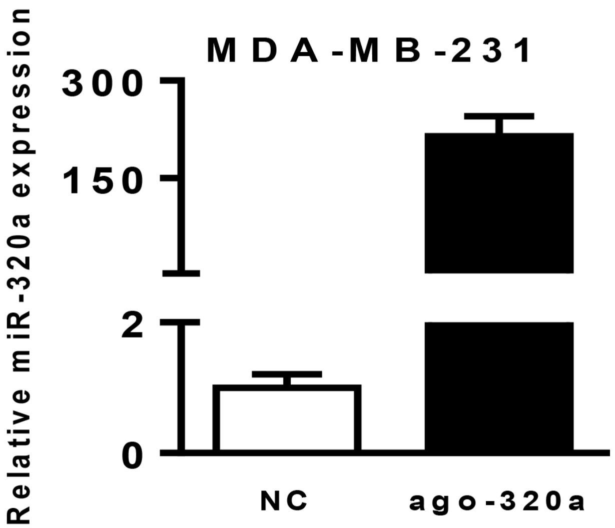

Supplementary Figure S4: MiR-320a expression after transfection with ago-miR-320a.

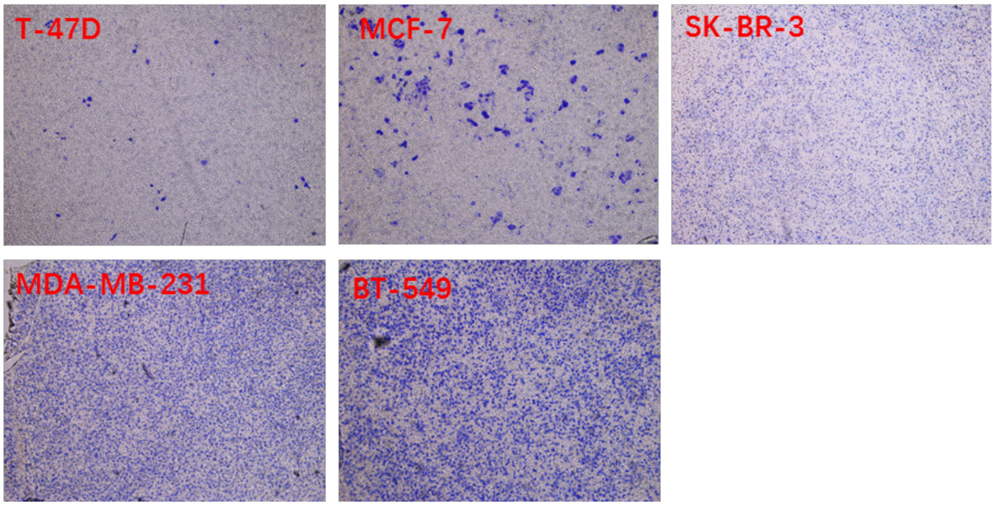

Supplementary Figure S5: Representative images of the transwell assay on migration abilities of five breast cancer cell lines (T-47D, MCF-7, SK-BR-3, BT-549, MDA-MB-231). We have performed migration assay to detect their migration abilities. Cells were seeded in a 24-well transwell plate with 1×10^5 cells in each chamber. After 24 h, cells passed through the membrane were stained with crystal violet. The results indicated that the migration abilities of T-47D and MCF7 cells were very weak.

Supplementary Table S1: PCR primers for luciferase reporter assay

Gene/Plasmid (length)	Primer	Sequence
GAPDH (220bp)	Forward	gacccccttcattgacctcaac
	Reverse	cttctccatggtggtgaaga
MTDH (153bp)	Forward	aaatgggcggactgttgaagt
	Reverse	ctgttttgcactgctttagcat
VDAC1 (184bp)	Forward	ccagcttcacactaatgtg
	Reverse	gttgttcactttagccgag
YWHAZ (138bp)	Forward	tgcaatgatgtactgtctc
	Reverse	tttcttgtcatcaccagcg
plucGRB2-3'UTRwt (514bp)	Forward	cacaactcgagtgcttccatggcttcctgag
	Reverse	aaggatcccaactctccctgcttcctgg
plucHLTF-3'UTRwt (1859bp)	Forward	cacaactcgagacacgtcttctaaaaggggca
	Reverse	aaggatccggaagttttcattccagtggct
plucMTDH-3'UTRwt (1431bp)	Forward	cacaactcgaggcagttgacacttgtgtatggc
	Reverse	aaggatccagcaacgcatctctgcatct
plucVDAC1-3'UTRwt (514bp)	Forward	cacaactcgagtgcagcatagctaccttcaga
	Reverse	aaggatcccacatccaccttctccaccc
plucYWHAZ-3'UTRwt (479bp)	Forward	ccctcgagctttgtttaatgtcttatg
	Reverse	cgggatccaacttacagttaatgctac
pcDNA3.1-MTDH (1952bp)	Forward	ccggaattctattccactgcgtctccgc
	Reverse	ccgctcgagtttatggtggtgtccgcag
plucMTDH-3'UTRmut1 (1431bp)	Forward	aaactagagtcagagaccattaattgacattg
	Reverse	tggtctctgactctagtttttcattctcc
plucMTDH-3'UTRmut2 (1431bp)	Forward	ggccttattaatggagaccattcctaacaag
	Reverse	tggtctccattaataaggccagtcttcagc
plucVDAC1-3'UTRmut (514bp)	Forward	tgattgtcgaaaaagatgttacccttccagaggta
	Reverse	atctttttcgacaatcaactttaacctggagggct
plucYWHAZ-3'UTRmut (479bp)	Forward	caactactttctctactggtattcatgtaaat
	Reverse	taccagtagagaaagtagttgaaaagtccatt

Supplementary Table S2: Potential target sites of wild-type and mutated sequences of miR-320a

Gene	Sequence
hsa-miR-320a	3′-AGCGGGAGAGUUGGGUCGAAAA-5′
GNB2L1 wt 14-20nt	5′-AAGUUUAUGGCAGAGCUUUAC-3′
hsa-miR-320a	3′-AGCGGGAGAGUUGGGUCGAAAA-5′
GRB2 wt 1232-1239nt	5′-GAGGCUUUAAGCUCCAGCUUUA-3′
hsa-miR-320a	3′-AGCGGGAGAGUUGGGUCGAAAA-5′
HLTF wt 302-308nt	5′-UAUCUUCCAAGUACUCAGCUUUU-3′
hsa-miR-320a	3′-AGCGGGAGAGUUGGGUCGAAAA-5′
MTDH wt 924-931nt	5′-AAAACUAGAGUCAGACAGCUUUA-3′
MTDH mut1	5′-GACCAUU
hsa-miR-320a	3′-AGCGGGAGAGUUGGGUCGAAAA-5′
MTDH wt 1501-1507nt	5′-GCCUUAUUAAUGGACAGCUUUC-3′
MTDH mut2	5′-GACCAUU-3′
hsa-miR-320a	3′-AGCGGGAGAGUUGGGUCGAAAA-5′
VDAC1 wt 137-144nt	5′-GGUUAAAGUUGAUUCAGCUUUA-3′
VDAC1 mut	5′-GUCGAAA-3′
hsa-miR-320a	3′-AGCGGGAGAGUUGGGUCGAAAA-5′
YWHAZ wt 1014-1020nt	5′-CUACUUUCUCUA...CAGCUUUU-3′
YWHAZ mut	5′-CUGGUAUU-3′

Schematic of the predicted binding sequences between miR-320a and potential target mRNA. The pluc-3′UTR-reporter constructs containing the wild-type (wt) or *mutated* (mut) sequences (underlined).
